# Navigation programs relevant for African American men with prostate cancer: a scoping review protocol

**DOI:** 10.1186/s13643-022-01993-6

**Published:** 2022-06-14

**Authors:** Nynikka R. Palmer, Ashley Nicole Smith, Brittany A. Campbell, Ghilamichael Andemeskel, Peggy Tahir, Tisha M. Felder, Barbara Cicerelli

**Affiliations:** 1 Division on General Internal Medicine at Zuckerberg San Francisco General Hospital, University of California San Francisco, 1001 Potrero Avenue, UCSF mailbox 1364, San Francisco, CA 94143 USA; 2 University of California San Francisco, 1450 3rd Street, San Francisco, CA 94143 USA; 3 UCSF Library, University of California San Francisco, 530 Parnassus Ave, San Francisco, CA 94143 USA; 4 College of Nursing, University of South Carolina, 1601 Greene Street, Room 620, Columbia, SC 29208 USA; 5 Zuckerberg San Francisco General Hospital, 995 Potrero Ave, Building 80, Room 8000N Lower Level, San Francisco, CA 94110 USA

**Keywords:** Patient navigation, Prostate cancer, African American, Scoping review

## Abstract

**Background:**

The excess incidence and mortality due to prostate cancer that impacts African American men constitutes the largest of all cancer disparities. Patient navigation is a patient-centered healthcare system intervention to eliminate barriers to timely, high-quality care across the cancer continuum and improves health outcomes among vulnerable patients. However, little is known regarding the extent to which navigation programs include cultural humility to address prostate cancer disparities among African American men. We present a scoping review protocol of an in-depth examination of navigation programs in prostate cancer care—including navigation activities/procedures, training, and management—with a special focus on cultural context and humility for African American men to achieve health equity.

**Methods:**

We will conduct comprehensive searches of the literature in PubMed, Embase, Web of Science, and CINAHL Complete, using keywords and index terms (Mesh and Emtree) within the three main themes: prostate cancer, patient navigation, and African American men. We will also conduct a search of the gray literature, hand-searching, and reviewing references of included papers and conference abstracts. In a two-phase approach, two authors will independently screen titles and abstracts, and full-text based on inclusion/exclusion criteria. All study designs will be included that present detailed data about the elements of navigation programs, including intervention content, navigator training, and/or management. Data will be extracted from included studies, and review findings will be synthesized and summarized.

**Discussion:**

A scoping review focused on cultural humility in patient navigation within the context of eliminating disparities in PCa care among African American men does not yet exist. This review will synthesize existing evidence of patient navigation programs for African American prostate cancer patients and the inclusion of cultural humility. Results will inform the development and implementation of future programs to meet the unique needs of vulnerable prostate cancer patients in safety net settings.

**Systematic review registration:**

PROSPERO 2021 CRD42021221412

## Background

Prostate cancer (PCa) is the second most common cancer among men in the United States of America (USA) [1]. The greatest cancer disparity is the gap in PCa incidence, aggressiveness, and mortality impacting African American men. African American men have a 76% higher incidence [1, 2], are more likely to be diagnosed with aggressive disease, and have more than twice the mortality due to PCa compared with White men [2–4]. In part, this is due to disparities in their receipt of definitive treatment [4, 5] and the fact that they experience greater treatment delays compared with White men [6, 7]. It is also no coincidence that African American men are over-represented in the low-resource healthcare settings where PCa disparities in the quality of care are most pronounced (e.g., public hospitals/clinics, “safety net”) [8–11]. Furthermore, poor communication [12, 13], implicit bias, and well-founded medical mistrust given historical context and personal experiences have an impact on variability in quality of care [14–16].

Patient navigation is an evidence-based intervention that can help reduce racial/ethnic disparities in cancer care [17, 18]. Launched in 1995 in Harlem, New York, patient navigation was developed by Dr. Harold Freeman to reduce health disparities among low-income African American women with an abnormal breast screening [19, 20]. Since its first use, researchers and healthcare professionals have broadened the practice across the cancer continuum to include cancer prevention, detection, diagnosis, treatment, and survivorship [21]. Navigation has been shown to eliminate disparities in delays in diagnostic resolution [17, 22–25] and treatment initiation and adherence [17, 26, 27], improve patient-centered communication, care coordination [28], and clinical outcomes [29].

The National Cancer Institute (NCI) implemented the Patient Navigation Research Program to address the need for standardization of navigation programs across health systems [30] and defines patient navigation as support and guidance offered to vulnerable persons with abnormal screening or a cancer diagnosis, with the goal of overcoming barriers to timely and effective diagnosis and treatment [30]. Notably, navigators are well-positioned to provide and improve cancer care within the context of cultural humility given their role as liaison between patients and providers [31]. Cultural humility takes a learning-oriented approach (curiosity and self-reflection over mastery) to working with people from diverse cultural backgrounds and emphasizes recognition of patients’ cultural perspectives as equally valid, and critical reflection on how systemic issues and power dynamics impact health care [32–34]. Honoring patients’ cultural values and lived experiences can shift power dynamics and make patients feel more welcomed and open to engage in healthcare [35]. Patient navigation programs can enact cultural humility through navigator training that emphasizes cultural sensitivity, openness, and appreciation of diverse perspectives of the patient population, and challenges within the healthcare system that disadvantage certain groups, including racism and implicit bias [36]. Navigation programs can also be culturally tailored, in which navigators are racially and linguistically matched to patients, as peers from one’s racial/ethnic group, age group, and/or gender that serve as sources of credible information. However, there is a dearth of published data on detailed components of patient navigation programs that include cultural context in content and training to meet the unique needs of specific racial/ethnic populations. While we identified an article that reviewed navigation from a culturally centered approach to address general cancer disparities [37] and another review article that examined metrics for cultural competency in navigation in lung cancer [38], to our knowledge, there are no scoping reviews focused on patient navigation in PCa care that examines cultural context for eliminating disparities among African American men.

## Aim and objectives

We aim to conduct a scoping review that examines navigation programs among African American men facing PCa from screening through survivorship, to uncover specific components and processes of navigation, including cultural humility to meet the unique needs of this underserved population, and how navigation is used to address disparities in quality of care. We will explore the following programmatic characteristics: (a) types of navigators—nurse, lay, peer, etc.; (b) navigators’ background and training; (c) managing navigators—who and how; (d) navigation activities/protocol; and (e) impact and outcomes measured. Results from this review will inform the development and implementation of an intervention protocol and training curriculum for peer navigation in safety net settings for African American men newly diagnosed with PCa.

## Methods/design

Our team will conduct a scoping review to identify published articles regarding navigation for African American men with PCa to explore details of cultural tailoring in navigation protocols, training of navigators, and measured outcomes. We chose a scoping review over a systematic review given our objective to examine the scope of relevant literature and inform practice of future patient navigation programs for African American men facing PCa [39]. This protocol was prepared in accordance with the Preferred Reporting Items for Systematic review and Meta-Analysis Protocol (PRISMA-P) statement for standardized reporting (see PRISMA-P checklist) [40] and the PRISMA extension for Scoping Reviews (PRISMA-ScR) [41]. This protocol was registered with the International Prospective Register of Systematic Reviews (PROSPERO; CRD42021221412) [42], although not required for scoping reviews [39].

### Eligibility criteria

All types of study designs will be included in this review. We will include studies of adult (18 years and older) males facing PCa. While we are focused on African American men, studies with other racial/ethnic groups will be considered for inclusion if at least 30% of participants were African American/Black men. Studies that describe or investigate navigation programs or interventions for PCa from screening through survivorship will also be eligible. We define navigation programs and interventions as any formal or informal, structured process designed to help patients overcome barriers to timely and effective health care [18, 43]. For example, navigation programs engage trained advocates (navigators) to interface with patients to identify and remove barriers to completing follow-up for cancer-related care, enhance patient-provider interactions, and reduce risk of lost to follow-up, including but not limited to keeping scheduled appointments, assessing understanding, arranging financial support, and adhering to treatment modalities [18, 44]. Studies must outline navigation intervention methods or specific details on program development, content, type of navigators and their training, management, implementation, or evaluation processes. We will assess outcome measures used in studies based on NCI Patient Navigation Research Methods [30], including but not limited to time to completion of diagnosis, time to initiation of primary treatment, patient satisfaction and quality of life, cost effectiveness, time to treatment completion, quality of care, navigator characteristics, and task and social network analysis. Studies will be excluded if the program or intervention (1) is designed for non-cancer conditions, (2) does not include at least 30% African American/Black adult men, (3) focuses solely on education or awareness, and (4) is focused on a population outside of the USA.

### Data sources and search strategy

To identify published literature, we will conduct a comprehensive search of the following electronic databases: PubMed, Embase, Web of Science, and CINAHL Complete. All publication dates will be included through June 2022 (our anticipated end search date). To identify gray literature (information produced outside traditional publishing and distribution channels—e.g., reports, white papers, etc.), the authors will hand search reference lists of included articles, conference abstracts, and conduct specialized Google searches. We will attempt to contact the corresponding author for conference abstracts found to get access to detailed information and results presented when no corresponding peer-reviewed publication is found. Abstracts and other gray literature will be excluded if no results or detailed information is obtained. Our biomedical research librarian (PT) developed search hedges along these three themes: PCa, peer navigation, and African American men. The peer navigation theme was expanded with terms encompassing community outreach, health disparities, community-based research, communication, education, health knowledge, decision making, and more. We used both keywords and index terms (Mesh and Emtree) in the construction of our searches. Complete search strategies for all databases are listed in Table 1. Our searches of the gray literature will comprise hand-searching and reviewing references from papers included in the final set of articles selected for our review. We will also review conference abstracts found in Embase for trends, background information, and the potential studies not yet published.

**Table 1:** Literature search strategy by databases

Database	Search strategy with no date restrictions
PubMed	(“Prostatic Neoplasms”[mesh] OR “prostate cancer” OR “prostate gland cancer” OR “prostatic cancer”) AND (“Patient Navigation”[mesh] OR “patient navigation” OR “patient navigator” OR “navigation program” OR “navigation programs” OR “navigation programme” OR “peer navigation” OR “peer navigator” OR “cancer navigation” OR “navigation system” OR “navigation intervention” OR “Health Education”[mesh] OR “health education” OR “cancer education” OR “cancer communication” OR “health advisor” OR “health advisors” OR “Community Health Workers”[mesh] OR “community health workers” OR “Health Promotion”[mesh] OR “health promotion” OR “outreach program” OR “outreach programs” OR “outreach programme” OR “outreach programmes” OR “Health Status Disparities”[mesh] OR “health status disparities” OR “healthcare disparities” OR “health care disparities” OR “health disparities” OR “Community-Based Participatory Research”[mesh] OR “research participation” OR “Health Knowledge, Attitudes, Practice”[mesh] OR “health attitudes” OR “health knowledge” OR “cancer knowledge” OR “medically underserved” OR “Early Detection of Cancer”[mesh] OR “early detection” OR “cancer detection” OR “cancer screening” OR “decision-making” OR “informed decision”) AND (“African Americans”[mesh] OR “African American” OR “African Americans” OR “black men” OR “black males”)
Web of Science	(“Prostatic Neoplasms” OR “prostate cancer” OR “prostate gland cancer” OR “prostatic cancer”) AND (“patient navigation” OR “patient navigator” OR “navigation program” OR “navigation programs” OR “navigation programme” OR “navigation programmes” OR “peer navigation” OR “peer navigator” OR “cancer navigation” OR “navigation system” OR “navigation intervention” OR “health education” OR “cancer education” OR “cancer communication” OR “health advisor” OR “health advisors” OR “community health workers” OR “health promotion” OR “outreach program” OR “outreach programs” OR “outreach programme” OR “outreach programmes” OR “health status disparities” OR “healthcare disparities” OR “health care disparities” OR “health disparities” OR “research participation” OR “health attitudes” OR “health knowledge” OR “cancer knowledge” OR “medically underserved” OR “early detection” OR “cancer detection” OR “cancer screening” OR “decision-making” OR “informed decision”) AND (“African American” OR “African Americans” OR “black men” OR “black males”)
Embase	('prostate cancer'/exp OR 'prostate cancer' OR 'prostate tumor'/exp OR 'prostate tumor' OR 'prostate gland cancer'/exp OR 'prostate gland cancer' OR 'prostatic neoplasms'/exp OR 'prostatic neoplasms') AND ('patient navigation'/exp OR 'patient navigation' OR 'patient navigator'/exp OR 'patient navigator' OR 'navigation program' OR 'navigation programs' OR 'navigation programme' OR 'navigation programmes' OR 'peer navigation' OR 'peer navigator' OR 'cancer navigation' OR 'navigation system'/exp OR 'navigation system' OR 'navigation intervention' OR 'health education'/exp OR 'health education' OR 'cancer education'/exp OR 'cancer education' OR 'cancer communication' OR 'health advisor' OR 'health advisors' OR 'community health workers'/exp OR 'community health workers' OR 'health auxiliary'/exp OR 'health auxiliary' OR 'health promotion'/exp OR 'health promotion' OR 'outreach program' OR 'outreach programs' OR 'outreach programme' OR 'outreach programmes' OR 'health disparity'/exp OR 'health disparity' OR 'research participation'/exp OR 'research participation' OR 'attitude to health'/exp OR 'attitude to health' OR 'health knowledge'/exp OR 'health knowledge' OR 'cancer knowledge' OR 'medically underserved'/exp OR 'medically underserved' OR 'early cancer diagnosis'/exp OR 'early cancer diagnosis' OR 'early detection' OR 'cancer detection'/exp OR 'cancer detection' OR 'cancer screening'/exp OR 'cancer screening' OR 'decision making'/exp OR 'decision making' OR 'informed decision making'/exp OR 'informed decision making' OR 'informed decision') AND ('african american'/exp OR 'african american' OR 'black men' OR 'black males')
CINAHL Complete	(“Prostatic Neoplasms” OR “prostate cancer” OR “prostate gland cancer” OR “prostatic cancer”) AND (“patient navigation” OR “patient navigator” OR “navigation program” OR “navigation programs” OR “navigation programme” OR “navigation programmes” OR “peer navigation” OR “peer navigator” OR “cancer navigation” OR “navigation system” OR “navigation intervention” OR “health education” OR “cancer education” OR “cancer communication” OR “health advisor” OR “health advisors” OR “community health workers” OR “health promotion” OR “outreach program” OR “outreach programs” OR “outreach programme” OR “outreach programmes” OR “health status disparities” OR “healthcare disparities” OR “health care disparities” OR “health disparities” OR “research participation” OR “health attitudes” OR “health knowledge” OR “cancer knowledge” OR “medically underserved” OR “early detection” OR “cancer detection” OR “cancer screening” OR “decision-making” OR “informed decision”) AND (“African American” OR “African Americans” OR “black men” OR “black males”)

Citations from the aforementioned databases will be collated, organized, and exported to the online Zotero bibliography management tool that can be shared among team members. Duplicates will be identified and removed. Remaining citations will move forward to the study selection process.

### Data screening and selection

**Fig. 1:**
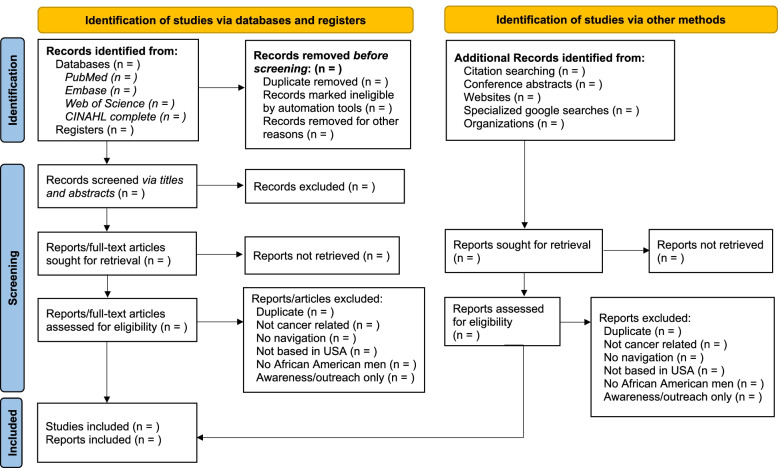
Outline of PRISMA 2020 flow diagram of the selection process. PRISMA: preferred reporting items for systematic reviews and meta-analyses

Three team members (ANS, BAC, and GA) will independently review and screen articles for the study selection process based on the inclusion and exclusion criteria in a two-step process. One reviewer (ANS) will screen all of the articles, and two additional reviewers (BAC and GA) will screen 50% of the articles each. First, two members will independently review article titles and abstracts, to assess eligibility of the articles against the inclusion criteria, and will note inclusion for further review or reason for exclusion. All marginally relevant articles and those that do not contain enough information to determine eligibility (e.g., no available abstract) will be retained. For phase II, two members (ANS, BAC, or GA) will independently review the full text of articles, noting inclusion or reason for exclusion based on our criteria. For both phases, the two reviewers will discuss decisions and any discrepancies to reach consensus. When consensus cannot be reached, outstanding conflicts will be resolved by a third reviewer (NRP or TMF) who has extensive experience in conducting reviews relevant to cancer care [45–50]. Reasons for exclusion will be documented, and all selection procedures will conform to the PRIMSA-ScR guidance [41]. Figure 1 presents a draft flow diagram that outlines our planned search and screening procedures.

### Data extraction

Prior to data extraction, three independent reviewers (ANS, BAC, and GA) will pilot test five included articles and refine our data extraction form based on their feedback. This will also enable us to develop clear instructions to ensure relevant results are extracted and all reviewers understand the data extraction process. We will build an electronic database for our data extraction form using REDCap (Research Electronic Data Capture)—a secure, web-based system supported by our institution for building and managing research projects, such as building databases, collecting data, and data validation [51]. Each reviewer (ANS, BAC, and GA) will complete data extraction on approximately 33% of the included articles (i.e., total number of included articles spilt amongst the three reviewers). To assess accuracy and comprehensiveness of data extraction, a second reviewer will conduct spot checks on at least 20% of the included articles. Any discrepancies found during data extraction will be resolved with team discussions and consensus, and a third reviewer (NRP or TMF) if needed. Based on the extent of disagreements identified, we will extend spot checks to a larger subset of studies.

We will extract data based on metrics set by the NCI Patient Navigation Research Program [30] and knowledge of existing patient navigation programs [23, 52–54] and include the full reference (author name, year of publication), study aims/objectives and design, conceptual framework/theory used, geographic location and setting, target population (participant characteristics, sample size, and phase of cancer continuum—e.g., screening, diagnosis, treatment, and survivorship), description of the intervention/navigation program (type of navigator, format, barriers addressed), data collection methods and outcomes, navigator training and management (recruitment, content, duration), and cultural context (see Table 2 for details). We will assess the inclusion of cultural context, guided by the PEN-3 model [55] and principles of cultural humility [32, 34, 56]. The PEN-3 cultural model places culture at the centerpiece of health beliefs, behaviors, and health outcomes, and framing solutions to health problems. PEN-3 consists of three primary domains and three PEN factors within each: (1) cultural identity [person, extended family, neighborhood], (2) relationships and expectations [perceptions, enablers, and nurturers], and (3) cultural empowerment [55] [positive, existential, and negative]. Cultural identity highlights the target audience or intervention points of entry (e.g., PCa survivor, nurse, community). Relationships and expectations highlight attitudes and beliefs of one’s social network that support or hinder health behaviors and health decisions. Cultural empowerment highlights cultural beliefs and practices that have positive, neutral/harmless, or negative health consequences. The PEN-3 model will guide the extraction of key words related to integrating culturally relevant factors to develop navigation programs—for example, how programs were shaped and messages clarified. Additionally, some concepts necessary for cultural humility include, but are not limited to, creating a safe environment that is respectful and nurturing; integrating humanities curricula (e.g., history of medicine, navigation, lessons learned, etc.); collaborating with community-based/public health experts; and understanding the role of language in patient-provider relationships [34]. Data extraction will include an iterative process, constant comparison, and synthesis of data to identify and categorize common themes that emerge that are aligned with the PEN-3 model and other relevant cultural aspects. For reconciliation of missing data in included studies, we will attempt to contact the corresponding author.

**Table 2:** Example data elements for data extraction

Category	Data elements
A. Reference	• Author(s) name(s) • Year of publication • Title of article
B. Study information	• Aims/objectives • Study design/type of study • Conceptual framework/theory used • Geographic location and setting • Study period (time frame)
C. Target population	• Participant characteristics • Sample size (intervention and control) • Cancer continuum phase (screening – survivorship)
D. Intervention/navigation program	• Name, objective, and description • Type and title of navigator • Format, location, and delivery methods • Duration and dose • Barriers addressed • Actions taken (e.g., referrals, accompaniment, etc.)
E. Data collection methods/measurements	• Outcomes (e.g., quality of life, satisfaction, etc.) • Follow-up period • Results and impact
F. Navigator training and management	• Recruitment and eligibility/qualifications of navigators • Location, format, and learning strategy • Content, materials • Duration and dose • Key learning points
G. Cultural context	• PEN-3 cultural model application/findings • Cultural humility factors • Themes identified

### Assessment of methodological quality

Two reviewers (ANS, BAC, or GA) will independently assess study quality and risk of bias using the Mixed Methods Appraisal Tool (MMAT) [57]—a tool that includes one set of items for critical appraisal of methodological quality designed for systematic reviews of mixed studies (quantitative, qualitative, mixed methods). Each item of the MMAT is rated on a categorical scale (yes, no, and cannot tell) and then “yes” will be counted (1 point) to provide an overall score from rating the five criterion per study design. Scores will be converted to percentages to grade quality of evidence: (i) ≤50% represents low-quality evidence, (ii) 50–75% represents average-quality evidence, and (iii) 76–100% represents high-quality evidence. We will also complete a descriptive summary using MMAT criteria to be more informative of the criterion, as recommended by MMAT developers [57]. Researchers have reported that the MMAT was easy to use, comprehensive, quick, short, and accessible online [58]. The MMAT is a useful tool for assessment quality given the heterogeneity of study designs we will include in this scoping review. As noted in data screening and extraction, disagreements between to two reviewers (ANS, BAC, or GA) will be resolved through discussion to reach consensus, and if necessary, a third reviewer (NRP or TMF) will resolve persistent disagreements.

### Data analysis/synthesis

We will report this scoping review following the PRIMSA-ScR format, which includes a checklist of 27 essential items for transparent reporting [41, 59]. In anticipation of heterogenous, quantitative, qualitative, and observational data, we will tabulate and present data in a narrative format that reflects our study objectives. Data will be organized by categories of navigation at different stages of disease, description of navigation models and elements, cultural tailoring, description of navigation training and management, and measures of outcome and impact. We will also discuss implications for future research and practice implications to eliminate disparities in PCa care among African American men.

## Discussion

Culturally tailored patient navigation provides a viable solution to the unique systemic problems faced by African American/Black men with PCa within the healthcare system. Previous reviews have investigated patient navigation programs for underserved populations across the cancer care continuum, noting more research is needed beyond screening and diagnosis, and outside of breast and colorectal cancers [27]. Furthermore, few reviews having taken a culturally centered approach to identify and examine how navigation models shape programs within the context of cultural humility and tailoring [37]. Our review will comprehensively synthesize existing navigation programs culturally tailored for African American men with PCa, filling a contemporary gap in the literature. By reviewing navigation interventions across the cancer continuum, we offer insight into culturally informed and tailored designs, training methods, implementation, and the effectiveness of navigation programs. Results will provide guidance for those contemplating developing, testing, and implementing future interventions and real-world programs to improve access to high-quality cancer care and achieve health equity.

We will disseminate findings from this scoping review via institutional and community partnerships, local and national presentations, and a peer-reviewed publication. We plan to utilize findings to inform expansion of our public hospital’s current cancer navigation program, led by a team member (BC). We will prepare presentations for (a) patients and community members within our networks (e.g., our PCa support group and community network of PCa advocates), (b) health care providers engaged in PCa care (e.g., institutional PCa symposiums and cancer center meetings), and (c) local and national scientific meetings (e.g., Academy of Oncology Nurse & Patient Navigators Conference or the American Association for Cancer Research – The Science of Cancer Health Disparities). Similarly, we will share findings with cancer-relevant community-based initiatives, such as the San Francisco Cancer Initiative—a cross-sector coalition to reduce cancer disparities [60, 61]. We will also seek guidance from our research team’s advisory board of PCa survivors to enhance our proposed dissemination plan (e.g., how and where to disseminate review findings) and to develop a future peer navigation program. We also anticipate building upon our existing partnerships to guide opportunities for future collaborations with various community-based organizations, such as African American fraternities, for more wide-spread implementation of a navigation training manual and navigation protocol for PCa care among African American men. These efforts will enhance the development of future interventions and real-world navigation programs to achieve health equity.

## Data Availability

All data generated or analyzed during this study will be available in another published article.
